# Indication for surgical treatment in patients with adolescent idiopathic scoliosis – a critical appraisal and counter-point

**DOI:** 10.1186/1754-9493-7-21

**Published:** 2013-07-03

**Authors:** Keith R Bachmann, Ryan C Goodwin, Timothy A Moore

**Affiliations:** 1 Cleveland Clinic Foundation, Departhment of Orthopaedic Surgery, Cleveland, USA; 2 MetroHealth Medical Center, Departments of Orthopaedic Surgery and Neurosciences, Case Western Reserve University School of Medicine, Cleveland, USA

**Keywords:** Scoliosis, Surgery, Complications, Indications

## Abstract

In a recent letter to the editor (Patient Saf. Surg. 2013, 7:17), Weiss and Moramarco made the claim that surgical correction of idiopathic scoliosis is not supported based on the available literature citing no medical indication and high complication rates as compared to non-operative management. In this letter we show that there is a role for surgical treatment as the only predictable option to obtain correction of a curve and that the risk of complications with newer instrumentation does not approach 50% as cited by Weiss and Moramarco. We share the opinion with Weiss and Moramarco that a decision for surgery is not one to be made lightly, and should include options of purely observation, bracing, as well as surgery depending on the potential progression profile and conversations with the child and parents.

## Correspondence

Adolescent Idiopathic Scoliosis (AIS), defined as asymmetry on forward bending combined with a curve of at least 10º by Cobb technique of a standing radiograph of the spine with vertebral rotation diagnosed after the age of 10 years [1], while not common in the general population with an incidence around 2-3% [2], is a problem frequently addressed by orthopaedic surgeons and a focus of school screening programs. Treatment regimens for AIS have consisted of observation, bracing, and surgery for varying degree curves. In a recent Letter to the Editor, Weiss and Moramarco make the claim that scoliosis surgery is not medically necessary, cosmetic improvements achieved with surgery are not necessarily stable, surgical intervention should only be pursued in AIS patients with substantial psychological problems due to the deformity and that these psychological problems need to be documented in the medical record prior to pursuing any surgical correction [3]. We hope to highlight with this rebuttal there are indications other than medical necessity and substantial psychological problems to pursue operative correction and fusion in an adolescent idiopathic scoliosis population including minimization of deformity and improvement of cosmesis.

Initial long term studies of scoliosis that supported surgical intervention due to higher mortality rates, back pain, and psychosocial effects including low marriage rate [4,5] were found to lump together infant and juvenile cases of idiopathic scoliosis as well as neuromuscular causes. There are however natural history studies of AIS patients highlighted by the Iowa cohort originally collected by Rubinstein and Ponseti most recently reported by Weinstein et al at 50 year follow-up [6]. In this cohort they show a range of final curves of 23°-156° for untreated thoracic curves with a mean of 85°, 50°-155° with a mean of 90° for thoraco-lumbar curves as well as 15°-90° with a mean of 49° for lumbar curves. These numbers represented an average progression of 1° per year after skeletal maturity in those curves between 40°-50° at skeletal maturity. They go on to highlight an increase in back pain that does not cause disability in untreated scoliosis patients, and maintained function despite their deformity. In their discussion; however, they highlight that patients with untreated AIS can develop significant deformity and it is unclear whether a contemporary cohort would be as accepting of deformity as the patients in their study.

Lonstein commented on operative and nonoperative decision making in scoliosis [7]. In his section on natural history, he highlights that pulmonary function is reduced in thoracic curves with a direct correlation between increasing curve magnitude and decreasing vital capacity, more pronounced with curves greater than 100° although measured in curves >60°. He notes that there are no reproductive effects of scoliosis, the psychosocial effects are difficult to evaluate but rib prominence is a concern, although studies are highlighted with high percentage of patients who are self conscious about their appearances [8]. Lonstein ends his discussion on natural history and progression by commenting that “the cosmetic aspect of scoliosis must be borne in mind and should not be minimized”. These findings highlight that while there may not be a medical necessity in terms of increased mortality to addressing AIS, there are certainly plenty of reasons to pursue treatment.

In regards to the maintenance of correction achieved by surgical correction, Westrick and Ward recently reviewed 5 to 20 year surgical results in AIS [9] demonstrating average Cobb angle correction with segmental hooks to be 51.2%, and in 2 studies with sufficient follow up with pedicle screw instrumentation the average correction was 69.5%. They found the loss of correction in these two populations to be 6.5% and 3.4% respectively. This highlights the power of contemporary segmental instrumentation by pedicle screws in addressing the three-dimensional and rotational nature of the scoliosis deformity. Modern instrumentation can not only inhibit deformity progression as highlighted by Weinstein et al’s natural history study but actually reduce and maintain deformity correction. The reoperation rate, even in the group with the highest reoperation rate, was 11.9% in the Harrington rod group over 20 years. Westrick and Ward state from the available studies it is not clear that the improved radiologic outcomes of pedicle screw instrumentation correlate with enhanced function, self-image, or overall health. In a concluding statement that Weiss references, Westrick notes “Although surgery reliably arrests the progression of deformity, achieves permanent correction, and improves appearance, there is no medical necessity for surgery based on the current body of literature”. It is also noted in the conclusion that “the senior author continues to support and perform surgical instrumentation and fusion in adolescents with significant curves and cosmetic concerns to prevent late progression and avoid increased risk and less satisfactory correction associated with surgical intervention in the adult”. Also quoting from Westrick, “the surgeon must not underestimate the psychological indication that occurs when a patient is no longer able to cope with the deformity”.

Going back to Lonstein’s article, a discussion of decision making in adolescents covers many scenarios, focusing on placing the child in the context of their presenting curve magnitude, growth potential as a time period where curves are predicted to progress the most, and comes away with a few clear indications for surgery. These indications include an actively growing adolescent with a curve of greater than 45° to 50°, a brace treated adolescent who progresses to a curve greater than 40° to 45° and thoracic lordosis which was not touched upon by Weiss but can inhibit pulmonary function and negative sagittal balance. Adolescents with thoracic lordosis have more pain symptoms. While these are indications to discuss surgery as a treatment option it is noted from Weiss as well as many others that discussion need be placed in the appropriate context. The medical necessity of the surgery has diminished over time as the natural history of AIS has been separated from the earlier onset as well as neuromuscular and other associated varieties of scoliosis, but in our opinion this does not eliminate all causes or indications for surgical correction of AIS. There is still a documented risk of progression of the curve and deformity into adulthood, with some increased risk of diminished pulmonary function if not life threatening, and the cosmetic concerns associated with deformity in a modern society.

We commend authors like Dr. Weiss who cause us to take a step back and ensure our interventions are impacting our patients in a positive way despite their risks. Instrumented fusion is not free of risk, but is a reliable method of gaining and maintaining correction of deformity and halting future progression of the curve and should be given appropriate consideration by patients and families with adolescent idiopathic scoliosis. Ultimately, surgical correction for AIS is a procedure to improve quality of life and should be decided upon by the patient and the family after a proper and appropriate conversation has occurred regarding the natural progression of the diagnosis, risks of the surgery and alternatives based on the present data available.

## Abbreviations

AIS: Adolescent idiopathic scoliosis.

## Competing interests

KB has no competing interest. RG receives > $5,000 per year as a paid speaker or consultant to Stryker, Inc. TM specialty editor spine for Journal of Bone and Joint Surgery.

## Authors’ contributions

KB participated in the background research as well as drafting of the manuscript, RG participated in background research as well as editing of the manuscript, TM participated in conception of the letter, background research, and editing of the manuscript. All authors read and approved the final manuscript.

## Authors’ information

KB is a resident in orthopedic surgery at the Cleveland Clinic. He is planning on pursuing a career in pediatric orthopedic surgery.

RG is a fellowship trained pediatric orthopedic surgeon in practice at the Cleveland Clinic. His specialties include pediatric spine deformity and correction as well as pediatric hip deformity among others.

TM is a fellowship trained orthopaedic spine surgeon in practice at MetroHealth Medical Center. His specialty surgery includes spine trauma surgery, adult deformity surgery, and still maintains a practice in orthopaedic trauma surgery.
